# Endowed Research

**Published:** 1909-02-13

**Authors:** 


### Endowed Research.

In a recent issue we urged the waste of intellect and the waste of material which obtain in our homes of medicine owing to the lack of endowments calculated to free our best investigators from the clutches of routine and spade-work. It is, therefore, with particular interest that we read in the columns of a contemporary* an account of the Rockefeller Institute for Medical Research. This Institute is the pattern of which we dream. It is true that the Institute lacks one item which, in our view, is vital to highest efficiency—namely, a hospital attached to it. But it appears that this deficiency is shortly to be made good, and in other respects it seems to fulfil all the essentials of our ideal. It was founded in 1901 by Mr. John D. Rockefeller, in order to further medicine in its various branches by establishing laboratories and by awarding money grants to trained investigators in other laboratories. Its management is vested in a Board of Directors composed of men eminent in medicine, Dr. William H. Welch being President, Dr. Emmett Holt Secretary, and Dr. Christian A. Herter Treasurer. The Director and Chief of the Laboratories is Dr. Simon Flexner. The home of the Institute is a large building in New York, amply equipped for every kind of medical research. The subdivision of the work of the Institute into departments is as yet incomplete. At present there are three such departments, one pathological, one devoted to physiology and pharmacology, and one to chemistry, each having its own staff of expert workers of various grades. Interchange of opinion is facilitated by a common luncheon room, in which the whole staff of the Institute meets daily, while the needs of the body are served by a well-kept tennis court—no small asset to men in the prime of physical life whose work is in the nature of things entirely sedentary. The published results of investigations which have been aided by grants, or which have been carried out in the laboratories of the Institute are collected into volumes of about 300 pages. So far eight volumes of these "Studies from the Rockefeller Institute" have appeared, the work having been carried out, so far as the contributions of the Institute itself are concerned, in a temporary building, the erection of the permanent one having been but recently completed. These "Studies" are sent to the leading laboratories, libraries, and learned societies both in America and abroad.

To read of such things makes one's mouth water, and lays an undue tax on our sense of national pride. For we have millionaires enough amongst us, and millionaires, too, to whom generosity is no stranger; yet the strivings of science do not seem to touch them as they touch the millionaires of America. Perhaps we are biassed, and too prone to fancy that ours is "the only pebble on the beach," as the saying goes; but certainly Mr. Carnegie's libraries seem a poor counterblast to the Rockefeller Institute. However, we live in hope. There is no doubt that medicine is daily taking a bigger place in national life as people come to realise the truth of the old adage touching prevention and cure. We must stick to our business and continue to justify our claims, and so, perhaps, at long last, the public will cease to leave the muzzle on the ox that treads the corn.

* *New York Medical Record*, January 23, 1909.

